# A Tumor-Imaging Method Targeting Cancer-Associated Fibroblasts

**DOI:** 10.2967/jnumed.118.210435

**Published:** 2018-09

**Authors:** Anastasia Loktev, Thomas Lindner, Walter Mier, Jürgen Debus, Annette Altmann, Dirk Jäger, Frederik Giesel, Clemens Kratochwil, Philippe Barthe, Christian Roumestand, Uwe Haberkorn

**Affiliations:** 1 Department of Nuclear Medicine, University Hospital Heidelberg, Heidelberg, Germany; 2 Clinical Cooperation Unit Nuclear Medicine, German Cancer Research Center (DKFZ), Heidelberg, Germany; 3 Faculty of Biosciences, Heidelberg University, Heidelberg, Germany; 4 Department of Radiation Oncology, University Hospital Heidelberg, Heidelberg, Germany; 5 Clinical Cooperation Unit Radiation Oncology, German Cancer Research Center (DKFZ), Heidelberg, Germany; 6 Department of Medical Oncology, National Center for Tumor Diseases (NCT), Heidelberg, Germany; 7 Centre de Biochimie Structurale, Université de Montpellier, CNRS, INSERM, Montpellier, France; and; 8 Translational Lung Research Center Heidelberg (TLRC), German Center for Lung Research (DZL), Heidelberg, Germany

**Keywords:** PET, radiopharmaceuticals, FAP, activated fibroblasts, small molecule, tumor

## Abstract

The tumor stroma, which accounts for a large part of the tumor mass, represents an attractive target for the delivery of diagnostic and therapeutic compounds. Here, the focus is notably on a subpopulation of stromal cells, known as cancer-associated fibroblasts, which are present in more than 90% of epithelial carcinomas, including pancreatic, colon, and breast cancer. Cancer-associated fibroblasts feature high expression of fibroblast activation protein (FAP), which is not detectable in adult normal tissue but is associated with a poor prognosis in cancer patients. **Methods:** We developed an iodinated and a DOTA-coupled radiotracer based on a FAP-specific enzyme inhibitor (FAPI) and evaluated them in vitro using uptake, competition, and efflux studies as well as confocal microscopy of a fluorescence-labeled variant. Furthermore, we performed imaging and biodistribution studies on tumor-bearing animals. Finally, proof of concept was realized by imaging patients with ^68^Ga-labeled FAPI. **Results:** Both FAPIs showed high specificity, affinity, and rapid internalization into FAP-expressing cells in vitro and in vivo. Biodistribution studies on tumor-bearing mice and on the first cancer patients demonstrated high intratumoral uptake of the tracer and fast body clearance, resulting in high-contrast images and negligible exposure of healthy tissue to radiation. A comparison with the commonly used radiotracer ^18^F-FDG in a patient with locally advanced lung adenocarcinoma revealed that the new FAP ligand was clearly superior. **Conclusion:** Radiolabeled FAPIs allow fast imaging with very high contrast in tumors having a high stromal content and may therefore serve as pantumor agents. Coupling of these molecules to DOTA or other chelators allows labeling not only with ^68^Ga but also with therapeutic isotopes such as ^177^Lu or ^90^Y.

See an invited perspective on this article on page 1412.

Tumor growth and spread are determined not only by cancer cells but also by the nonmalignant constituents of the malignant lesion, which are subsumed under the term *stroma*. The stroma may represent over 90% of the mass in tumors with a desmoplastic reaction, such as breast, colon, and pancreatic carcinoma. In particular, a subpopulation of fibroblasts called cancer-associated fibroblasts is known to be involved in tumor growth, migration, and progression. Therefore, these cells represent an attractive target for diagnosis and antitumor therapy.

A distinguishing feature of cancer-associated fibroblasts is expression of fibroblast activation protein (FAP), a type II membrane-bound glycoprotein belonging to the dipeptidyl peptidase 4 family. FAP has both dipeptidyl peptidase and endopeptidase activity (1). The endopeptidase activity distinguishes FAP from the other members of the dipeptidyl peptidase 4 family. The substrates identified thus far for the endopeptidase activity are denatured type I collagen, α_1_-antitrypsin, and several neuropeptides (2–4). FAP has a role in normal developmental processes during embryogenesis and in tissue modeling. On adult normal tissues, it is expressed only insignificantly or not at all. However, high expression occurs in wound healing, arthritis, atherosclerotic plaques, fibrosis, and in more than 90% of epithelial carcinomas (5–7).

The presence of FAP in cancer-associated fibroblasts in many epithelial tumors and the fact that overexpression is associated with a worse prognosis in cancer patients led to the hypothesis that FAP activity is involved in cancer development, cancer cell migration, and cancer spread. Therefore, targeting of this enzyme for imaging and endoradiotherapy can be considered a promising strategy for detecting and treating malignant tumors. We addressed this task by developing a small-molecule radiopharmaceutical based on a FAP-specific inhibitor and were able to show specific uptake, rapid internalization, and successful imaging of tumors in animal models and tumor patients. A first comparison with the commonly used radiotracer ^18^F-FDG in a patient with locally advanced lung adenocarcinoma revealed that the new FAP ligand was clearly superior.

## MATERIALS AND METHODS

### Compound Synthesis and Radiochemistry

Two radiotracers based on a FAP-specific inhibitor (8) were synthesized. A radioiodine-labeled FAP-specific enzyme inhibitor (FAPI), FAPI-01, was obtained via an organotin stannylated precursor, which was prepared through palladium catalyzed bromine/tin exchange. FAPI-02, a precursor for the chelation of radiometals, was synthesized in 5 steps (Supplemental Fig. 3; supplemental materials are available at http://jnm.snmjournals.org). Radioiodinations of the stannylated precursor were performed with peracetic acid. For chelation with ^177^Lu and ^68^Ga, the pH of the reaction mixture was adjusted with sodium acetate and heated to 95°C for 10 min. Stability in human serum was analyzed by precipitation and radio–high-performance liquid chromatography analysis of the supernatant. More information on the synthesis chemistry and analytics are provided in the supplemental material.

### In Vitro Characterization of FAPI Derivatives

In vitro binding studies were performed using the human cancer cell lines BxPC3, Capan-2, MCF-7 (Sigma Aldrich Chemie GmbH), and SK-LMS-1 (ATCC), as well as the stably transfected human FAP cell line HT-1080-FAP, the murine FAP-expressing human cell line HEK-muFAP, and the CD26-expressing human cell line HEK-CD26 (obtained from Stefan Bauer, NCT, Heidelberg, Germany). For fluorescence internalization experiments, cells were seeded on coverslips and stained with FAPI-02-Atto488 and 4′,6-diamidino-2-phenylindole for cell nucleus staining. Images were acquired on a laser-scanning confocal microscope using a ×63 oil immersion objective. Radioligand binding studies were performed using HT-1080-FAP cells. The radiolabeled compound was added to the cell culture and incubated for intervals ranging from 10 min to 24 h. Competition experiments were performed by simultaneous exposure to unlabeled (10^−5^ to 10^−9^ M) and radiolabeled compound for 60 min. For efflux experiments, radioactive medium was removed after incubation for 60 min and replaced by nonradioactive medium for intervals ranging from 1 to 24 h. For internalization experiments, surface-bound activity was removed by incubating the cells with 1 M glycine-HCl buffer for 10 min. The radioactivity was measured using a γ-counter, normalized to 1 million cells, and calculated as percentage injected dose (%ID). Detailed information on the cell lines and assays is given in the supplemental material.

### PET Imaging and Biodistribution Analysis on Mice

For in vivo experiments, BALB/c *nu/nu* mice (Charles River) were subcutaneously inoculated with HT-1080-FAP or Capan-2 cells. PET imaging was performed up to 140 min after intravenous injection of 4 nmol of ^68^Ga-FAPI-02 (10 MBq) per mouse using an Inveon small-animal PET scanner (Siemens). Images were reconstructed iteratively using 3-dimensional maximum a posteriori ordered-subset expectation maximization and were converted to SUV images. Quantification was done using a region-of-interest technique and expressed as mean SUV. For organ distribution of ^177^Lu-FAPI-02 (∼1 MBq/mouse), the animals (*n* = 3 for each time point) were sacrificed after indicated time points from 30 min to 24 h. The distributed radioactivity was measured in all dissected organs and in blood using a γ-counter (Cobra Autogamma; Packard). The values are expressed as %ID per gram of tissue (%ID/g). Additional information on the animal experiments can be found in the supplemental material.

### Clinical PET/CT Studies

Diagnostic imaging of 3 patients with ^68^Ga-FAPI-02 PET/CT for medical reasons was performed under the conditions of the updated declaration of Helsinki (section 37, unproven interventions in clinical practice) and in accordance with the German Pharmaceuticals Law (section 13, 2b). The tracer was injected intravenously (20 nmol, 222–312 MBq), and images were obtained 10 min, 1 h, and 3 h later. One of the patients was also imaged using ^18^F-FDG PET/CT (1 h after receiving 358 MBq). The images were obtained on a Biograph mCT Flow PET/CT scanner (Siemens Medical Solutions) using the following parameters: a 5-mm slice thickness, an increment of 3–4 mm, a soft-tissue reconstruction kernel, and CARE Dose4D (Siemens Medical Solutions). Immediately after the CT component had been acquired, whole-body PET was performed in 3 dimensions (matrix, 200 × 200) in FlowMotion (Siemens Medical Solutions) at a rate of 0.7 cm/min. The emission data were corrected for random, scatter, and decay events. Images were reconstructed using ordered-subset expectation maximization with 2 iterations and 21 subsets and were Gauss-filtered to a transaxial resolution of 5 mm in full width at half maximum. Attenuation was corrected using the low-dose nonenhanced CT data. SUVs were quantitatively assessed using a region-of-interest technique. All patients gave written informed consent. The evaluation was approved by our institutional ethical review board (approval S-016/2018).

## RESULTS

### FAPI-01 Selectively Targets Human and Murine FAP

To analyze the binding properties of ^125^I-FAPI-01 to its target protein, radioligand binding assays were performed on the 4 human cancer cell lines and on the 3 cell lines transfected with human or murine FAP or with the closely related membrane protein dipeptidyl peptidase 4/CD26. Both murine FAP and CD26 have been shown to have a high homology to human FAP (muFAP: 90% identity and 94% similarity on an amino acid level; CD26: 52% identity and 71% similarity, with high structural resemblance) (9).

**FIGURE 1.**
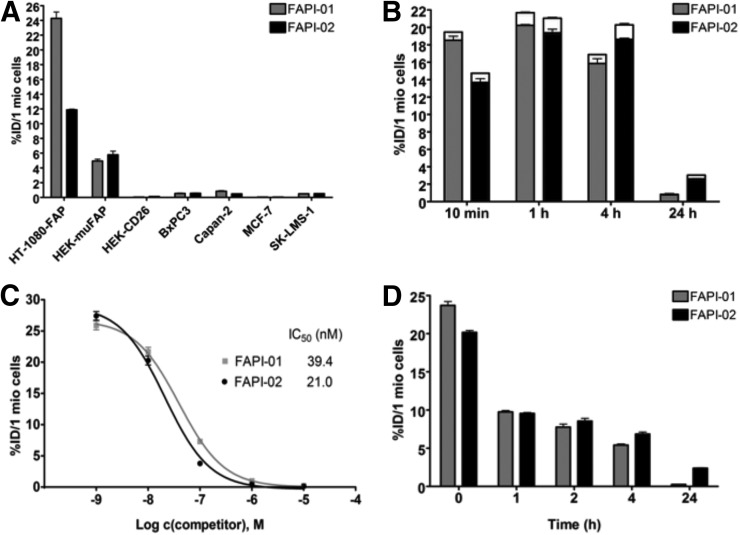
(A) Binding of radiolabeled FAPI-01 and FAPI-02 to the 4 human cancer cell lines and to the HT-1080-FAP, HEK-muFAP, and HEK-CD26 cell lines after 60 min of incubation. (B) Internalization of radiolabeled FAPI-01 and FAPI-02 into HT-1080-FAP cells after incubation for 10 min to 24 h. Internalized fraction is gray or black, and extracellularly bound fraction is white. (C) Competitive binding of radiolabeled FAPI-01 and FAPI-02 to HT-1080-FAP cells after adding increasing concentrations of unlabeled FAPI-01 and Lu-FAPI-02. (D) Efflux kinetics of FAPI-01 and FAPI-02 after 1 h of incubation of HT-1080-FAP cells with radiolabeled compounds, followed by incubation with compound-free medium for 1–24 h. All data are %ID normalized to 1 million (mio) cells.

As seen in Figure 1A, ^125^I-FAPI-01 showed no significant binding to the FAP-negative cancer cell lines but targeted human and murine FAP-expressing cells with high affinity (half-maximal inhibitory concentration for human FAP, 39.4 nM). Additionally, no substantial binding to CD26-expressing cells was observed (0.05% ± 0.01%), proving that ^125^I-FAPI-01 selectively targets FAP. This characteristic is of particular importance because CD26 is highly expressed in a variety of normal tissues, including kidneys, liver, and small intestine. To avoid a high background signal due to unspecific CD26 binding, high selectivity of the ligand to FAP is of great advantage, resulting in optimal image quality.

### FAPI-01 Rapidly Internalizes in FAP-Expressing Cells but Shows Time-Dependent Efflux and Robust Deiodination

Cell-based internalization assays demonstrated rapid uptake of ^125^I-FAPI-01 into the cells (Fig. 1B). After 10 min of incubation, 95% of the total bound fraction was located intracellularly (total, 19.70% ± 0.28%). Over 4 h, only a marginal decrease in activity was observed (total, 17.00% ± 0.40%, of which 94% was internalized).

Iodine-labeled compounds often show a time-dependent enzymatic deiodination. This was also observed for ^125^I-FAPI-01, resulting in low intracellular radioactivity of this compound after longer incubation times (3.25% ± 0.29% after 24 h; Supplemental Fig. 5). Deiodination can be minimized by reduction of deiodinase activity after lowering of the temperature to 4°C, resulting in an increased radioactivity of 26.66% ± 1.59% after 24 h (Supplemental Fig. 5).

### FAPI-02 Shows Enhanced Binding and Uptake to Human FAP as Compared with FAPI-01

To avoid rapid loss of ^125^I-FAPI-01 activity due to enzymatic deiodination, a nonhalogen derivative, FAPI-02, was designed, in which the FAP-binding moiety is chemically linked to the chelator DOTA. In addition to the resulting enhanced stability, this modification offers the possibility of easily incorporating either diagnostic or therapeutic radionuclides, allowing FAPI-02 to be used as a theranostic compound. Like its iodized analog, ^177^Lu-FAPI-02 specifically binds to human and murine FAP-expressing cells (half-maximal inhibitory concentration for human FAP, 21 nM) without addressing CD26 (0.13 ± 0.01 %ID; Fig. 1A). ^177^Lu-FAPI-02 internalizes rapidly into FAP-expressing cells (20.15 ± 1.74 %ID after 60 min, of which 96% is internalized; Fig. 1B), showing more stable and higher uptake rates over time. Unlike the high binding of ^125^I-FAPI-01 after 10 min of incubation, only 5% of the ^125^I-FAPI-01 activity remained after 24 h. In contrast, 34% of the initial radioactivity of ^177^Lu-FAPI-02 was detected after 24 h of incubation. Efflux experiments demonstrated that ^177^Lu-FAPI-02 was eliminated significantly more slowly than ^125^I-FAPI-01, showing retention of 12% of the originally accumulated radioactivity after 24 h (^125^I-FAPI-01, 1.1 %ID after 24 h; Fig. 1D).

**FIGURE 2.**
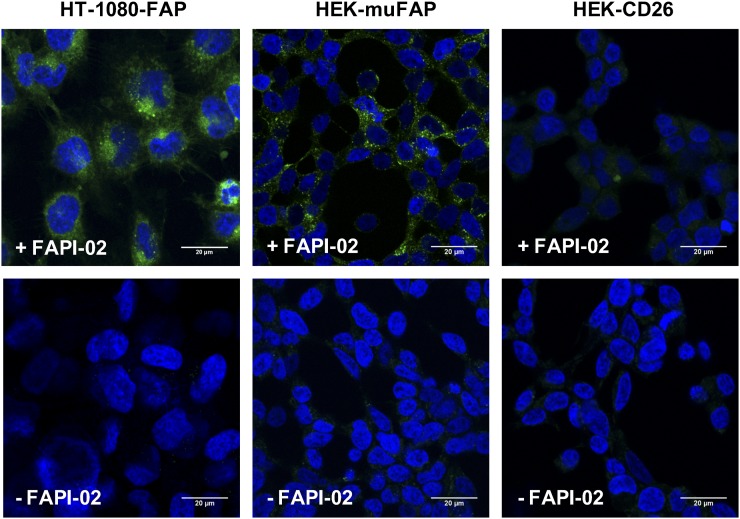
Internalization of FAPI-02 into HT-1080-FAP cells, HEK-muFAP cells, and HEK-CD26 cells after incubation for 2 h. Blue indicates 4′,6-diamidino-2-phenylindole, and green indicates FAPI-02-Atto488.

Robust internalization of FAPI-02 into human and murine FAP-expressing cells was confirmed by fluorescence laser-scanning microscopy. To this end, HT-1080-FAP and HEK-muFAP cells were stained with a fluorescently labeled FAPI-02 derivative (FAPI-02-Atto488) for 1–2 h. As shown in Figure 2, the compound was completely internalized and accumulated inside FAP-expressing cells, whereas no uptake was detectable in FAP-negative HEK-CD26 cells.

### FAPI-02 Accumulates in Human FAP-Expressing and FAP-Negative Xenografts by Recruitment and Activation of Mouse Fibroblasts

**FIGURE 3.**
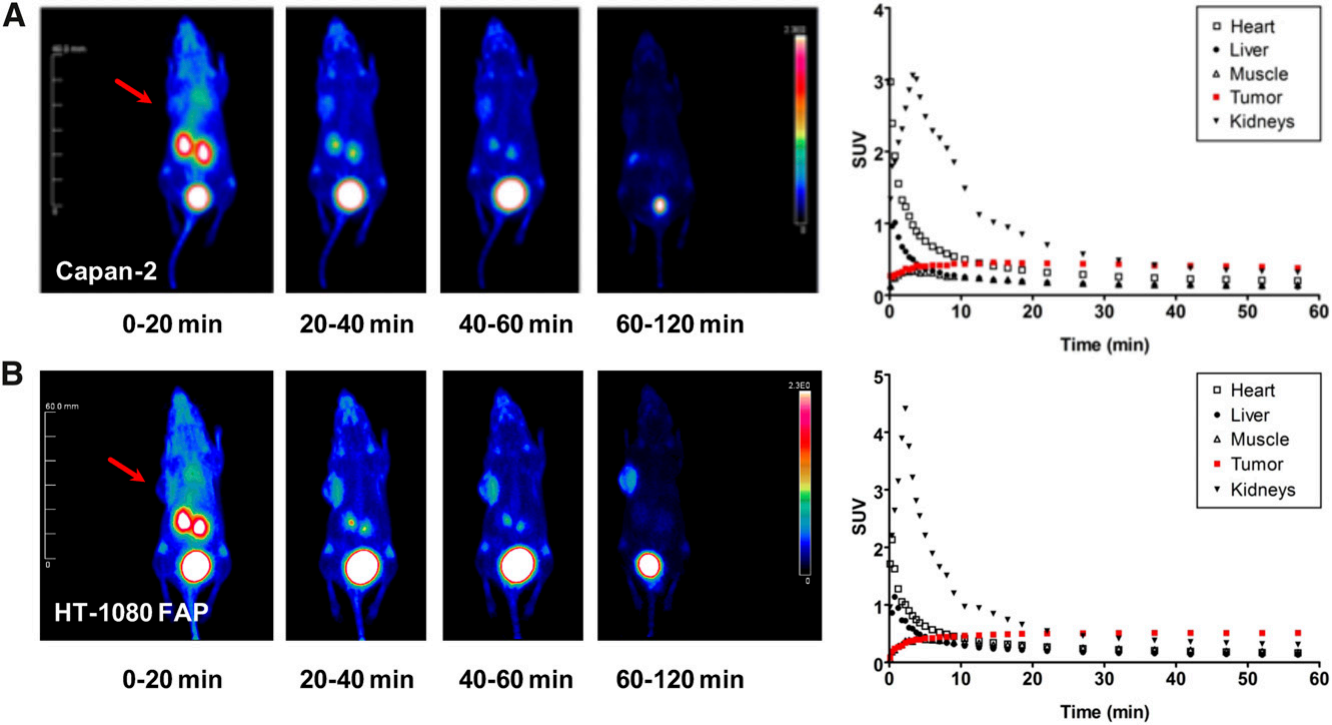
^68^Ga-FAPI-02 PET in mice bearing FAP-negative (Capan-2) (A) and human FAP-expressing (HT-1080-FAP) (B) xenografts. Images were obtained at the indicated times after injection and show rapid uptake in tumor (arrows), no accumulation in noncancerous tissue, and rapid elimination via kidneys and bladder. Quantification of PET images shows solid clearance from cardiovascular system and constant uptake into tumor.

**FIGURE 4.**
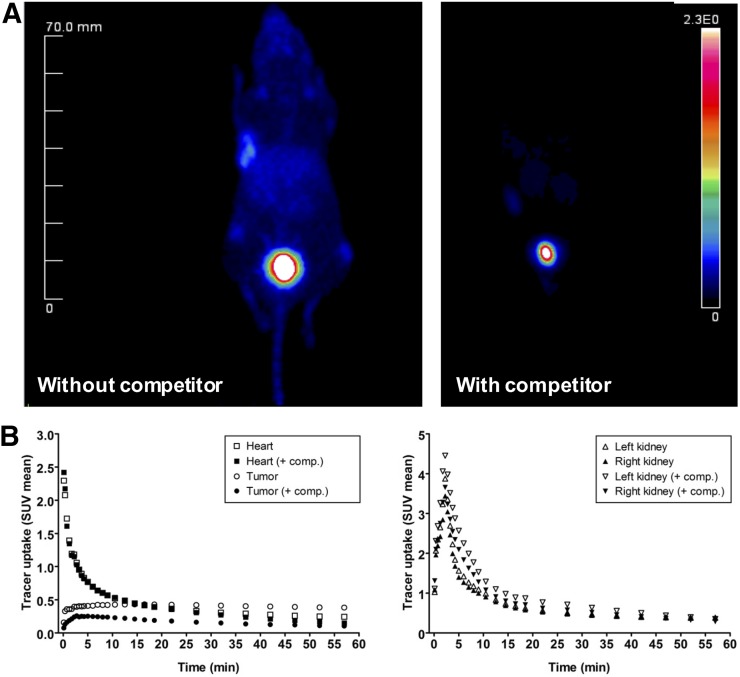
(A) Blocking of ^68^Ga-FAPI-02 tumor accumulation by coadministration of 30 nmol of unlabeled FAPI-02 in mice bearing HT-1080-FAP tumors. (B) Time–activity curves for ^68^Ga-FAPI-02 in selected organs after administration with and without unlabeled FAPI-02 as competitor.

Tumor accumulation of ^68^Ga-FAPI-02 was assessed by small-animal PET imaging of mice bearing xenografts from both human FAP-expressing and human FAP-negative tumor cells. In both cases, the radiotracer was taken up rapidly by tumor and the uptake was maintained for at least 140 min (Figs. 3A and 3B). At the same time, ^68^Ga-FAPI-02 showed negligibly low unspecific binding and was quickly eliminated from the blood, predominantly via the kidneys and bladder, resulting in low background activity and beneficial tumor-to-organ ratios. Simultaneous administration of unlabeled FAPI-02 as competitor resulted in a complete absence of radioactivity in the tumor, demonstrating the specificity of the radiotracer to its target protein (Fig. 4). Interestingly, high tumor uptake of ^68^Ga-FAPI-02 was observed in mice bearing FAP-expressing (HT-1080-FAP) as well as FAP-negative (Capan-2) cancer cell lines because of recruitment and activation of mouse fibroblasts. The pharmacokinetic characteristics of the radiotracer, calculated from PET data using a 2-tissue-compartment model according to a previously published method (10), are given in Supplemental Table 1.

**FIGURE 5.**
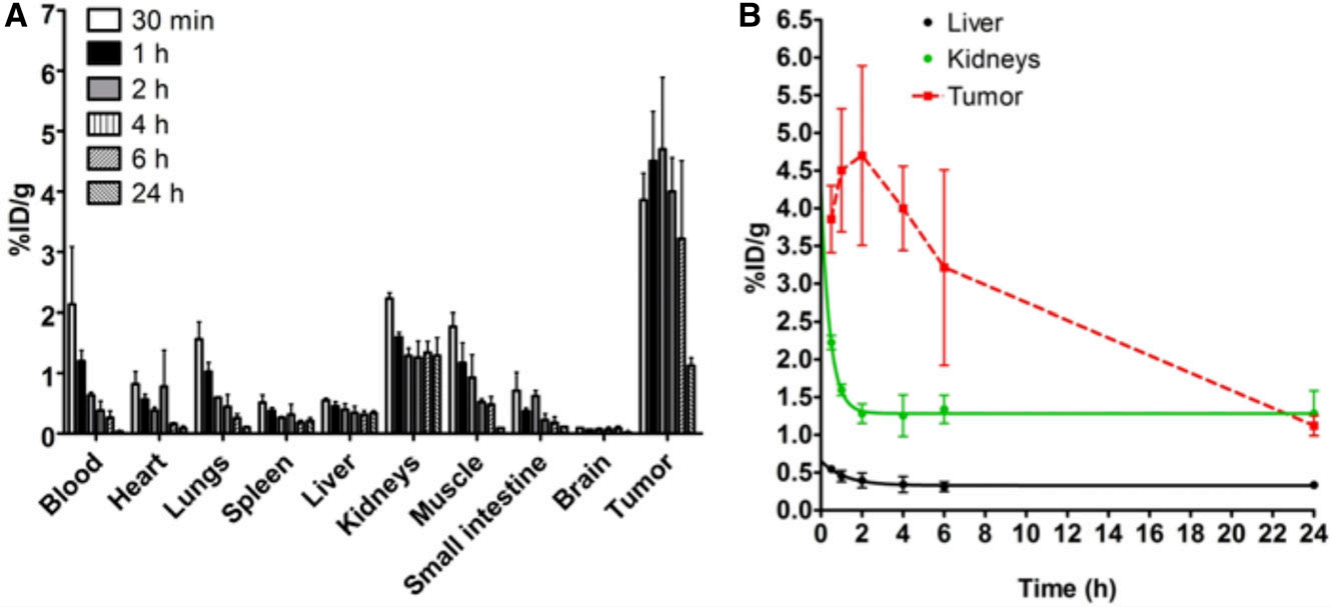
(A) Ex vivo biodistribution of ^177^Lu-FAPI-02 after administration to mice bearing HT-1080-FAP tumors. Tumor uptake is highest after 2 h (4.7 %ID/g). (B) Pharmacokinetic profile of ^177^Lu-FAPI-02 up to 24 h after administration.

These observations were confirmed using ^177^Lu-FAPI-02 in a biodistribution study, proving rapid tumor accumulation in both human FAP-expressing and human FAP-negative tumors and very low activity in all other organs (Fig. 5; quantified uptake values are given in Supplemental Table 2), resulting in beneficial tumor-to-organ ratios (Supplemental Fig. 6).

### FAPI-02 Rapidly Accumulates in Breast and Pancreatic Cancer Metastases in Humans

**FIGURE 6.**
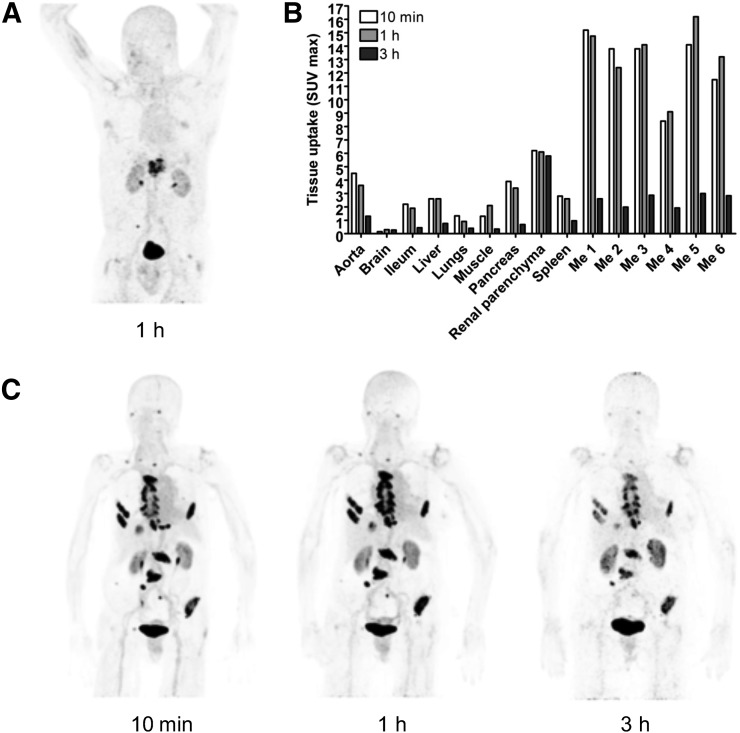
PET/CT maximum-intensity projections of patient with metastasized pancreatic cancer (A) and patient with breast cancer (C). (B) Maximum uptake of ^68^Ga-FAPI-02 at 10 min, 1 h, and 3 h after administration to breast cancer patient. Me = metastases.

**FIGURE 7.**
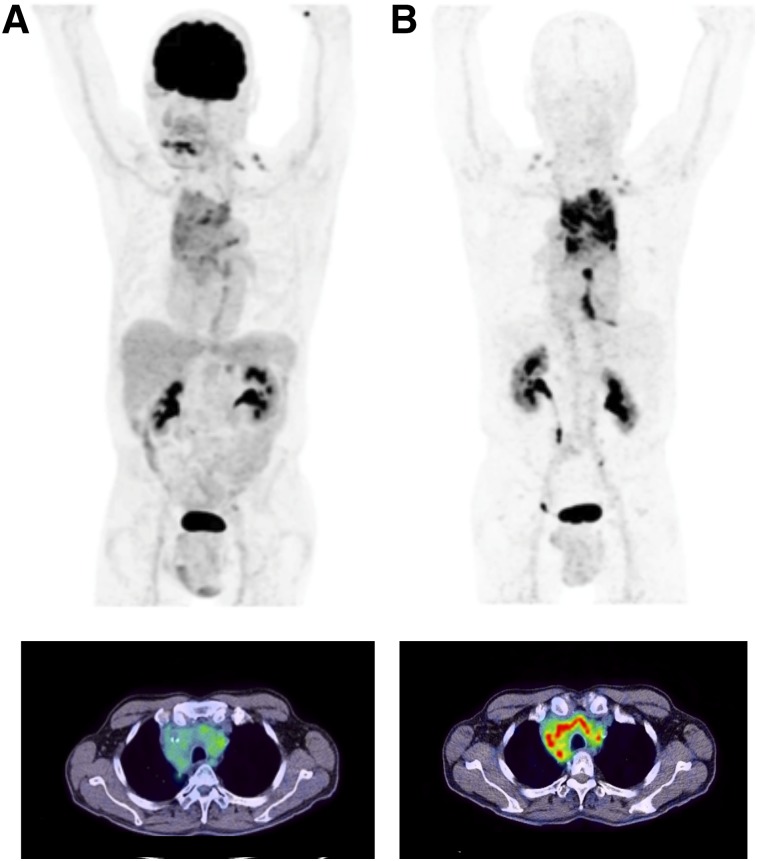
PET/CT maximum-intensity projections (top) and transaxial views (bottom) 1 h after administration of ^18^F-FDG (A) and ^68^Ga-FAPI-02 (B) to patient with locally advanced lung adenocarcinoma. ^68^Ga-FAPI-02 is seen to selectively accumulate in FAP-expressing tissue and to be significantly higher than ^18^F-FDG in malignant lesions. Unlike ^18^F-FDG, ^68^Ga-FAPI-02 shows no uptake in brain, spleen, or liver.

Diagnostic PET/CT scans were performed 10 min, 1 h, and 3 h after intravenous administration of ^68^Ga-FAPI-02 in a patient with metastasized breast cancer, a patient with metastasized lung cancer, and a patient with metastasized pancreatic cancer. In all 3 patients, a robust accumulation of the tracer was observed in the primary tumor and in lymph node and bone metastases, with a maximum SUV of 13.3. In contrast, tracer uptake in normal tissue was very low (Fig. 6; Supplemental Table 3). The radioactivity cleared rapidly from the bloodstream and was excreted predominantly via the kidneys, resulting in high-contrast images. Comparative imaging in the patient with locally advanced lung adenocarcinoma revealed an obvious advantage of ^68^Ga-FAPI-02 over the commonly used PET tracer ^18^F-FDG. As shown in Figure 7, ^68^Ga-FAPI-02 had higher uptake in metastatic lesions and lower background activity, leading to higher contrast and better visibility of the lesions. In contrast to ^18^F-FDG, which accumulates strongly in cells with high glucose consumption, such as the brain, ^68^Ga-FAPI-02 selectively targets tissues in which FAP is expressed.

## DISCUSSION

Reliable diagnosis of primary tumors, metastatic lesions, and affected lymph nodes is of the upmost importance in planning effective therapy, including disease staging and treatment choice. For this purpose, imaging techniques represent indispensable tools for the assessment of many cancer types. Because of its high diagnostic accuracy and the possibility of evaluating both anatomic and physiologic details, combined PET/CT is the method of choice for modern tumor diagnostics (11). However, in contrast to anatomic imaging techniques such as MRI or CT alone, PET/CT requires the use of radiotracers with a high affinity to targeted structures whose expression is higher in tumors than in normal tissues. An ideal tracer should specifically bind to its target protein to ensure reliable differentiation between cancerous and healthy tissue as well as a low background signal, resulting in high-contrast images. Affinity and specificity become even more important if the radiotracer represents a theranostic compound—that is, offers the possibility of being loaded with both diagnostic and therapeutic nuclides, thus facilitating and improving targeted and personalized treatment. Regarding potential applications of the tracer for therapeutic purposes, high target specificity ensures reduced side effects, a consideration that is especially important for the protection of radiation-sensitive tissue such as bone marrow, reproductive organs, and digestive organs.

With that in mind, we aimed to develop a theranostic tracer targeting cancer-associated fibroblasts, which form a major component of the tumor stroma. They are known to play a critical role in tumor growth, migration, and progression and are genetically more stable than cancer cells, therefore being less susceptible to the development of therapy resistance. In contrast to normal fibroblasts, cancer-associated fibroblasts express particular proteins that can be used as tumor-specific markers. Among these is the membrane protein FAP, which is broadly expressed in the microenvironment of, and thus enables targeting of, a variety of tumors. Such tumor types include pancreas, breast, and lung cancer, which account for a large part of the entirety of solid tumors.

By focused chemical modification of a small-molecule enzyme inhibitor with high affinity to its target protein (8), we developed the radiotracers FAPI-01 and FAPI-02. Both compounds demonstrated specific binding to human and murine FAP, showing rapid and almost complete internalization without addressing the closely related protein dipeptidyl peptidase 4/CD26. Because iodinated molecules undergo an enzymatic deiodination with efflux of free iodine, longer incubation times result in a lower intracellular radioactivity. On this account, FAPI-02 was designed to have the FAP-binding moiety chemically linked to the chelator DOTA, resulting in a theranostic compound with favorable pharmacokinetic and biochemical properties. ^177^Lu-FAPI-02 is eliminated significantly more slowly than ^125^I-FAPI-01, with retention of 12% of the originally accumulated radioactivity after 24 h (^125^I-FAPI-01, 1.1%). FAPI-02 rapidly internalizes into FAP-expressing cells and shows high tumor uptake rates in both tumor-bearing mice and patients with metastasized epithelial carcinomas. In contrast, there is no accumulation in normal tissue, and clearance from the blood system is rapid, resulting in high-contrast images. The robust internalzation into both human and murine FAP-expressing cells was confirmed by confocal microscopy using fluorescence-labeled FAPI-02. In contrast to the first-generation FAP-antibody F19, which has high affinity to its target protein without being internalized, FAPI-02 shows complete intracellular uptake after 1 h of incubation. The mechanism of internalization after FAP binding has been studied by Fischer et al. using FAP antibody fragments and DyLight 549 antimouse antibody (Thermo Fisher Scientific) in SK-Mel-187 cells. Incubation at 37°C led to internalization of the FAP–antibody complexes (12). As with our small molecule, the internalization process was fast, with almost complete internalization. Colocalization of the antibody fragments with a marker for early endosomes was observed after 20 min, and colocalization with a marker for late endosomes and lysosomes was observed after 40 min. Antigen-binding fragment–mediated FAP internalization was suppressed by an inhibitor for dynamin-dependent endocytosis, indicating that endocytosis occurs by a dynamin-dependent mechanism.

FAPI-02 is quickly eliminated from the organism by renal clearance without being retained in the renal parenchyma. In contrast to ^18^F-FDG, which accumulates strongly in cells with high glucose consumption, including inflammatory tissue and the brain, ^68^Ga-FAPI-02 is selectively taken up in tissues where its target protein is expressed. The fact that there is no or very low ^68^Ga-FAPI-02 uptake in all normal organs, especially the brain and the liver, opens new perspectives for the detection of malignant lesions in these regions based on images with a very high contrast. Additionally, FAP has also been shown to be expressed by rheumatoid myofibroblastlike synoviocytes in patients with rheumatoid arthritis and osteoarthritis (6), atherosclerosis (1), and fibrosis (13,14), as well as in ischemic heart tissue after myocardial infarction (15,16). These observations suggest a broader application of FAPI-02 as an imaging tracer for indications in nononcologic diseases.

FAP imaging has already been realized using antibodies and an inhibitor molecule (17–20). For detection of atherosclerotic plaques, the boronic acid–based FAP inhibitor MIP-1232 has been used (20,21). Radioiodinated MIP-1232 showed high accumulation in FAP-expressing SK-Mel-187 xenografts in vitro. However, binding to endarterectomized tissue was similar in plaques and normal arteries, indicating that atherosclerosis imaging using this compound may be difficult (20). Imaging of rheumatoid arthritis has been performed in animal models using the antibody 28H1 labeled with ^111^In, ^89^Zr, or ^99m^Tc, with the inflamed joints showing high uptake that correlated with the arthritis score (18,19).

The anti-FAP antibody sibrotuzumab has been labeled with ^131^I and used for therapy in patients with metastasized FAP-expressing carcinomas (22,23). The antibody showed a slow elimination from liver, spleen, and other normal organs that was consistent with blood pool clearance. High uptake was seen in metastatic lesions larger than 1.5 cm in all patients, frequently 2 d after administration (22). Imaging results were improved using the SPECT technique, detecting lesions down to 1 cm in diameter (23). The optimal time for imaging was 3–5 d after injection of the radiolabeled antibody. However, these imaging studies were performed with ^131^I-labeled antibodies using planar imaging or SPECT for tumor detection. The use of a β-emitting isotope with a high-energy γ-emission requires the use of high-energy collimators and thick crystal detectors, which highly limit image resolution. Along with the slow clearance of antibodies resulting in an enhanced background signal, this limitation inevitably leads to difficulty in detecting small lesions. This restriction can be overcome using small molecules such as MIP-1232 or the FAPI molecules developed by our laboratory and using PET instead of SPECT. In this respect, the limiting factor for the detection of tumor lesions is the degree of FAP expression within the tumor. This degree largely depends on the number of activated fibroblasts, that is, the percentage of stromal content, or the number of FAP molecules per fibroblast, which may be determined by the microenvironment. Because tumor growth exceeding 1–2 mm essentially requires the formation of a supporting stroma (15), visualization of small lesions in the range of 3–5 mm should be possible using FAPI-PET/CT.

As with any other targeted approach, FAPI-02 achieves optimal results only in tissues with a sufficiently high FAP expression, which is known to be rather heterogeneous in different cancer types and patients. Besides breast, colon, and pancreatic cancer, which are excellent candidates for FAPI imaging, further analyses have to explore whether tumor entities such as lung cancer, head and neck cancer, ovarian cancer, and hepatomas represent favorable targets.

Also, the fact that FAP expression was demonstrated in wound healing and fibrotic tissue should be kept in mind when interpreting radiologic findings, to properly evaluate which patients are likely to benefit from a potential FAPI-02 therapy. Given the ability to use either diagnostic or therapeutic nuclides, FAPI-02 allows simple stratification of the appropriate patient cohort. Either way, it is already clear that FAPI-02 represents an ideal lead candidate for the development of a targeted radiopharmaceutical. Because of its high target affinity, rapid tumor internalization, and fast body clearance, it is already ideally suited for tumor imaging. For therapeutic applications, future work has to concentrate on prolongation of the tumor retention time. This may be done by modification of the binding moiety or the linker or by use of different chelators (24).

## CONCLUSION

Radiolabeled FAPIs allow fast imaging with very high contrast in tumors having a high stromal content and may therefore serve as pantumor agents. Coupling of these molecules to DOTA or other chelators allows labeling not only with ^68^Ga but also with therapeutic isotopes such as ^177^Lu or ^90^Y.

## DISCLOSURE

This work was funded in part by grant 13N 13341 from the Federal Ministry of Education and Research. Philippe Barthe and Christian Roumestand were supported by the French Infrastructure for Integrated Structural Biology (FRISBI) (ANR-10-INBS-05). Uwe Haberkorn, Anastasia Loktev, Thomas Lindner, and Walter Mier were named in a patent application (EP 18155420.5) for quinolone-based FAP-targeting agents for imaging and therapy in nuclear medicine. No other potential conflict of interest relevant to this article was reported.
