# Supplementary material for: A Tumor-Imaging Method Targeting Cancer-Associated Fibroblasts
Source: J Nucl Med. 2018 Sep;59(9):1423–9. doi: 10.2967/jnumed.118.210435 (PMC6126438; doi:10.2967/jnumed.118.210435)
Supplement: Supplementary file 1 [file jnm210435SupplementalData.pdf]

Reagents

All solvents and non-radioactive reagents were obtained in reagent grade from ABCR (Karlsruhe, Germany), Sigma-Aldrich (München, Germany), Acros Organics (Geel, Belgium) or VWR (Bruchsal, Germany) and were used without further purification. Atto 488 NHS-ester was obtained from AttoTec (Siegen, Germany). 2,2',2''-(10-(2-(4-nitrophenyl)oxy)-2-oxoethyl)-1,4,7,10-tetraazacyclo-dodecane-1,4,7triyl)triacetic acid (DOTA-PNP) was synthesized following the protocol of Mier et al. (*1*). The intermediates 6-methoxyquinoline-4-carboxylic acid (**7**), 5-bromoquinoline-4-carboxylic acid (**3**) and (*S*)-1-(2-aminoacetyl)pyrrolidine-2-carbonitrile 4-methylbenzenesulfonate were synthesized following the protocols of Jansen *et al.* (*2*). The substance (*S*)-*N*-(2-(2-cyanopyrrolidin-1-yl)-2-oxoethyl)-5-bromoquinoline carboxamide was synthesized by a modified HBTU amidation protocol.

Compound Synthesis

Scheme 1 depicts the initial synthesis of FAPI-01 which was achieved by performing a Br/Li-exchange with *n*-butyllithium at 5-bromoquinolie-4-carboxylic acid (**3**) and quenching with elemental iodine to obtain iodoquinoline **4**. This compound was coupled to the Gly-Pro-CN fragment by HBTU/HOBt-activation to provide non-radioactive reference material of FAPI-01 (**1**).

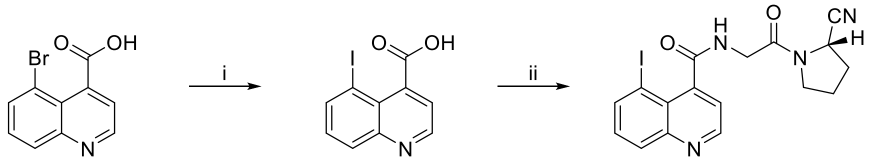

3 **4** **1**

SUPPLEMENTAL FIGURE 1. Synthesis of non-radioactive FAPI-01. i) *n*BuLi, then I_2, THF; ii) HBTU/HOBt, DIPEA, H-Gly-Pro-CN, DMF.

For the synthesis of radioactive FAPI-01 (**1***), the stannylated precursor **6** was obtained by palladium-catalyzed stannylation of inhibitor **5** in dioxane at 80°C (Scheme 2).

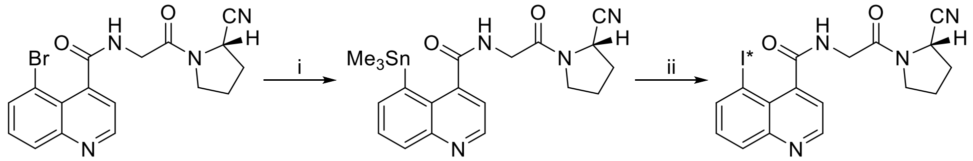

5 **6** **1***

SUPPLEMENTAL FIGURE 2. Synthesis of radioactive FAPI-01 via the stannylated precursor **4**. i) $(Me_3Sn)_2$; $(PPh_3)_2PdCl_2$; dioxane 80 °C; ii) I-125 or I-131; AcOOH; 1 M HCl; MeOH.

To enable radiolabeling by incorporation of radiometals, the chelator DOTA was chemically linked to the basic scaffold of the FAP-inhibitor. As shown by Jansen et al. (*2*), modifications at the 6-position of the quinoline-4-carboxylic acid are well tolerated without impairing target affinity and specificity. Therefore, a bifunctional linker was attached to the hydroxyl group of **8** via an ether linkage, leading way to the synthesis shown in Scheme 3. Ready available 1-bromo-3-chloropropane was chosen to create a spacer, which is unharmed during the saponification of the simultaneously formed ester bond at the end of the one-pot-process. Compound **9** was converted to the *N*-Boc protected quinolinecarboxylic acid **10** which was further coupled to H-Gly-Pro-CN by HBTU. Due to the high

hygroscopicity of the free amine, compound **11** was directly converted to FAPI-02 (**2**) after the Boc-removal, solvent exchange and neutralization of excess *p*-toluenesulfonic acid.

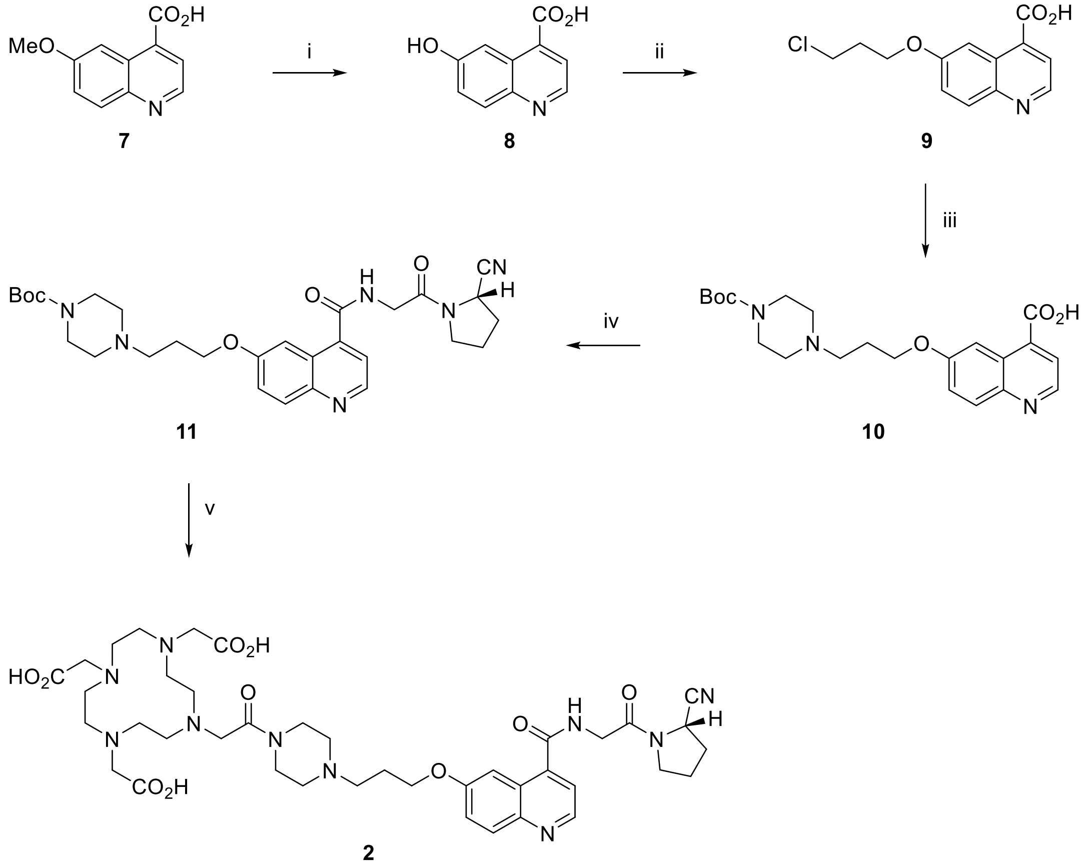

SUPPLEMENTAL FIGURE 3. Chemical synthesis of FAPI-02. i) aq. HBr 48%, 130 °C; ii) 1-bromo-3-chloropropane, Cs_2CO_3, DMF then 6 M NaOH; iii) 1-Boc-piperazine, KI, DMF; iv) HBTU/HOBt, DIPEA, H-Gly-Pro-CN, DMF; v) TosOH, MeCN, then DOTA-PNP, DIPEA, DMF.

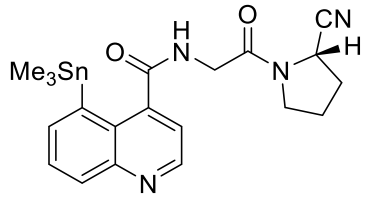

(*S*)-*N*-(2-(2-cyanopyrrolidin-1-yl)-2-oxoethyl)-5-trimethylstannylquinoline caboxamide (**6**)
3.88 mg (10.0 µmol) (*S*)-*N*-(2-(2-cyanopyrrolidin-1-yl)-2-oxoethyl)-5-bromoquinoline caboxamide, 20 µL (32 mg; 96 µmol) hexamethylditin and 0.75 mg (1.07 µmol) bis(triphenylphosphine)palladium(II) dichloride in 1 mL dry dioxane were stirred at 80 °C over night under an inert atmosphere. Volatiles were removed and the residue was taken up in 2 mL 50% acetonitrile/water and filtered through a SPE cartridge (sep-pak light C18, Waters) before HPLC-purification. 2.78 mg (5.90 µmol; 59%) of the product were obtained after freeze drying.
LC-MS R_t 14.77 min, m/z 473.0786 $[M(^{120}Sn)+H]^+$

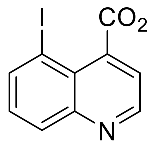

5-iodoquinolie-4-carboxylic acid (**4**)

5.42 mg (136 µmol) of sodium hydride suspension (60% in mineral oil) were added to a solution of 30.27 mg (120 µmol) 5-bromoquinolie-4-carboxylic acid (**3**) in 3 mL dry THF under Ar at 0°C. The ice bath was removed and the reaction mixture was cooled to -78 °C before 100 µL (160 µmol) nBuLi (1.6 m in hexanes) were added dropwise. After 15 min 64.71 mg (254 µmol) iodine in 2 mL THF were added dropwise and the reaction was stirred for 30 min at -78 °C before allowed to reach room temperature. After 1 h the reaction was quenched by addition of 1 mL 0.5 M $NaHCO_3$ and ca. 30 mg (170 µmol) sodium dithionite to remove excessive iodine. After the removal of THF under reduced pressure the mixture was acidified to pH 2 and extracted three times with ethyl acetate (25 mL). The combined organic phases were evaporated to dryness and purified by HPLC. 18.14 mg (60.7 µmol; 45%) of the title compound were obtained after freeze drying.

^{1}H NMR (500 MHz, DMSO-d6) 13.95 (br, 0.3H), 8.93 (s, 1H), 8.34 (d, J =7.2 Hz, 1H), 8.12 (d, J = 8.4 Hz, 1H), 7.60 (s, 1H), 7.52 (t, J = 7.9 Hz, 1H); **^{13}C NMR** (125 MHz, DMSO-d6) 168.8, 150.3, 148.8, 141.3, 130.6, 121.0, 109.5; **LC-MS** R_t 8.65 min, m/z 299.9383 $[M+H]^+$

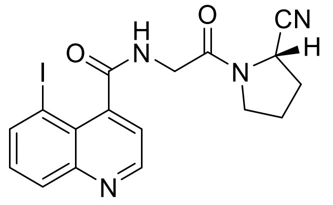

(*S*)-*N*-(2-(2-cyanopyrrolidin-1-yl)-2-oxoethyl)-5-trimethylstannylquinoline caboxamide (**1**; FAPI-01)

9.07 mg (23.9 µmol) HBTU in 50 µL DMF were added to a solution of 6.21 mg (20.8 µmol) 5-iodoquinoline-4-carboxylic acid, 7.45 mg (55.2 µmol) HOBt and 10 µL DIPEA in 50 µL DMF. After 15 min (29.9 µmol) (*S*)-1-(2-aminoacetyl)pyrrolidine-2-carbonitrile 4-methylbenzenesulfonate in 50 µL DMF were added. The reaction was quenched with 850 µL water and purified by HPLC. Freeze drying provides 6.86 mg (15.8 µmol; 76%) of the product.

^{1}H NMR (600 MHz, DMSO-d6) 9.06, 8.97, 8.33, 8.13, 7.56, 7.51, 4.81, 4.34, 4.06, 3.74, 3.56, 2.21, 2.17, 2.09, 2.05; **^{13}C NMR** (150 MHz, DMSO-d6) 167.1, 150.2, 148.8, 145.3, 141.5, 130.7, 125.3, 121.9, 119.3, 92.0, 46.3, 45.4, 42.1, 29.5, 24.9; **LC-MS** R_t 11.95 min, m/z 435.0102 $[M+H]^+$

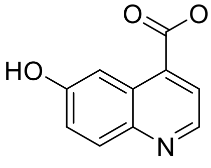

6-Hydroxyquinoline-4-carboxylic acid (**8**)

105 mg (477 µmol) of raw 6-methoxyquinoline-4-carboxylic acid (**7**) were dissolved in 3 mL of 48% hydrobromic acid in water. The solution was heated to 130 °C for 4 h. The solution was brought to a slightly basic pH with 6 M NaOH after reaching room temperature. 79.2 mg (419 µmol; 88%) of the product were obtained after by HPLC-purification and lyophilization.

^{1}H NMR (500 MHz, DMSO-d6) 13.65 (br, 0.6H) 10.24 (s, 1H), 8.78 (d, J = 4.4 Hz, 1H), 8.06 (d, J = 2.6 Hz, 1H), 7.95 (d, J = 9.1 Hz, 1H), 7.84 (d, J = 4.4 Hz, 1H), 7.37 (dd, J = 9.1, 2.6 Hz, 1H), **^{13}C NMR** (125 MHz, DMSO-d6) 167.7, 156.9, 146.5, 144.1, 133.4, 131.2, 126.2, 122.3, 122.6, 106.5; **LC-MS** R_t 6.66 min, m/z 190.0415 $[M+H]^+$

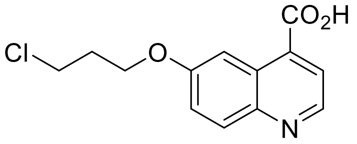

6-(3-chloro-1-propoxy)quinoline-4-carboxylic acid (**9**)

42.4 µL (67.4 mg; 430 µmol) 1-bromo-1-chloropropane were added to a suspension of 23.2 mg (123 µmol) 6-hydroxyquinoline-4-carboxylic acid (**8**) and 190 mg (1.38 µmol) potassium carbonate in 250 µL DMF and heated to 60 °C over night. The reaction mixture was cooled to room temperature, diluted with 500 µL water and 500 µL acetonitrile before 100 µL 6 M NaOH were added. The reaction mixture was directly purified via HPLC after the complete ester hydrolysis was accomplished. 26.45 mg (99.4µmol; 81%) of the product were obtained after lyophilization.

^{1}H NMR (500 MHz, DMSO-d6) 13.75 (br, 0.4H), 8.88 (d, J = 4.4 Hz, 1H), 8.19 (d, J = 2.0 Hz, 1H), 8.04 (d, J = 9.2 Hz, 1H), 7.94 (d, J = 4.4 Hz, 1H), 7.52 (dd, J = 9.2, 2.0 Hz, 1H), 4.24 (t, J = 5.95 Hz, 2H), 3.85 (t, J = 6.5 Hz, 2H), 2.27 (m, 2H); **^{13}C NMR** (125 MHz, DMSO-d6) 167.6, 157.5, 147.6, 144.8, 134.0, 131.2, 125.9, 122.7, 122.2, 104.5, 64.7, 41.9, 31.6; **LC-MS** R_t 11.46 min, m/z 266.0461 $[M+H]^+$

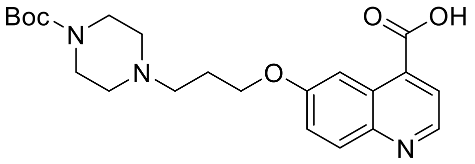

6-(3-(4-*tert*-butoxycarbonylpiperazin-1-yl)-1-propoxy)quinoline-4-carboxylic acid (**10**)

15.13 mg (56.9 µmol) of 6-(3-chloro-1-propoxy)quinoline-4-carboxylic acid (**9**), 55.43 mg (298 µmol) *N*-*tert*-butoxycarbonylpiperazine and 51.05 mg (30.8 µmol) potassium iodide were dissolved in 250 µL DMF. The reaction was shaken at 60 °C over night. The resulting suspension was diluted with 750 µL water before the product was purified by HPLC. After freeze drying 28.73 mg (54.3 µmol; 95%) of the product were obtained as the corresponding TFA-salt.

^{1}H NMR (500 MHz, D_2O) 8.93 (d, J = 5.5 Hz, 1H), 8.17 (d, J = 9.3 Hz, 1H), 7.94 (d, J = 5.5 Hz, 1H), 7.79 (dd, J = 9.3, 2,5 Hz, 1H), 7.65 (d, J = 2.5 Hz, 1H), 4.36 (t, J = 5.6 Hz, 2H), 4.27 (d, J = 13.55 Hz, 2H), 3.67 (d, J = 11.95 Hz), 3.47 (t, J = 15.5 Hz, 2 H), 3.27 (t, J = 12.7 Hz), 3.12 (td, J = 12.2, 2.65 Hz), 2.37 (m2 H), 1.47 (s, 9H); **^{13}C NMR** (125 MHz, D_2O) 155.5, 153.5, 149.0, 141.4, 134.4, 127.9, 126.6, 122.3, 118.4, 110.0, 105.1, 82.8, 65.5, 54.3, 51.5, 48.6, 40.7, 29.6, 27.4; **LC-MS** R_t 10.62 min, m/z 416.1997 $[M+H]^+$

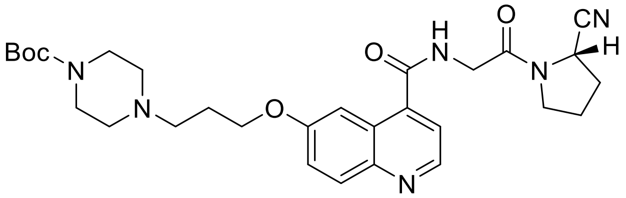

(*S*)-*N*-(2-(2-cyanopyrrolidin-1-yl)-2-oxoethyl)-6-(3-(4-*tert*-butoxycarbonylpiperazin-1-yl)-1-propoxy)quinoline-4-carboxamide (**11**)

9.43 mg (24.9 µmol) HBTU in 50 µL DMF were added to a solution of 10.56 mg (19.9 µmol) 6-(3-(4-*tert*-butoxycarbonylpiperazin-1-yl)-1-propoxy)quinoline-4-carboxylic acid (**10**), 5.38 mg (39.8 µmol)

HOBt and 10 µL DIPEA in 50 µL DMF. After 15 min (29.9 µmol) (*S*)-1-(2-aminoacetyl)pyrrolidine-2-carbonitrile 4-methylbenzenesulfonate in 50 µL DMF were added. The reaction was quenched with 850 µL water and purified by HPLC. Freeze drying provides 12.88 mg (19.4 µmol; 97%) of the title compound.

^{1}H NMR (500 MHz, DMSO-d6) 9.04 (d, J = 5.5 Hz, 1H), 8.24 (d, J = 9.6 Hz, 1H), 8.10 (d, J = 5.5 Hz, 1H), 7.89 (d, J = 2.3 Hz, 1H), 7.85 (dd, J = 9.6, 2.3 Hz, 1H), 4.84 (t, J = 6 Hz, 1 H), 4.46–4.36 (m, 4H), 4.26 (d, J = 12.0 Hz, 2H), 3.83 (m, 1H), 3.67 (m, 3H), 3.47 (t, J = 7.7 Hz, 2H), 3.27 (br, 2H), 3.11 (t, J = 11.5 Hz), 2.37 (m, 4H), 2.22 (m, 2H), 1.46 (s, 9H); **^{13}C NMR** (125 MHz, DMSO-d6) 168.6, 168.0, 159.4, 155.5, 147.7, 141.8, 135.1, 128.2, 127.5, 123.1, 120.0, 119.1, 104.7, 82.9, 66.0, 54.3, 51.5, 47.0, 46.3, 42.3, 29.4, 27.4, 24.7, 23.1; **LC-MS** R_t 11.81 min, m/z 551.2736 $[M+H]^+$

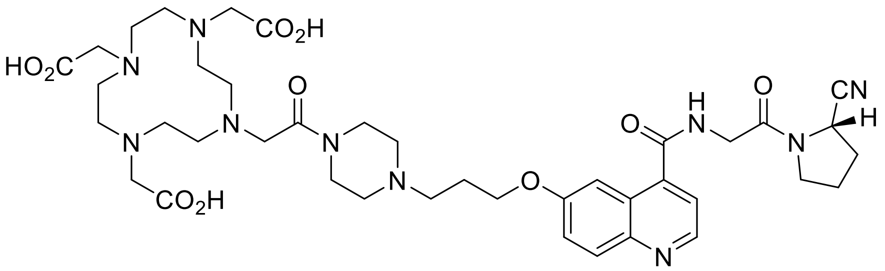

FAPI-02 (**2**)

4.85 mg (8.80 mmol) (*S*)-*N*-(2-(2-cyanopyrrolidin-1-yl)-2-oxoethyl)-6-(3-(4-*tert*-butoxycarbonyl-piperazin-1-yl)-1-propoxy)quinoline-4-carboxamide (**11**) were dissolved in 1 mL acetonitrile and 4.2 mg (22.0 µmol) 4-methylbenzenesulfonic acid monohydrate were added. The reaction was shaken at 45 °C over night, before volatiles were removed under reduced pressure. The residue was taken up in 190 µL dimethylformamide and 10 µL (7.3 mg; 72 µmol) triethylamine before 6.77 mg (12.9 mmol) of DOTA-*p*-nitrophenol ester were added. The reaction mixture was diluted with 1 mL water and purified by HPLC after shaking for two hours. 5.04 mg (6.02 µmol; 68%) were obtained after freeze drying.

^{1}H NMR (600 MHz, D_2O) 9.02, 8.23, 8.07, 7.87, 7.83, 4.85, 4.45, 4.41, 4.40, 4.39, 3.83, 3.67, 3.50, 3.49, 2.40, 2.38, 2.36, 2.26, 2.22, 2.16; **^{13}C NMR** (150 MHz, D_2O) 167.9, 159.1, 147.2, 141.8, 135.4, 127.9, 127.2, 119.8, 119.0, 104.5, 65.8, 54.1, 46.8, 46.1, 42.1, 29.2, 24.5, 23.0: **LC-MS** R_t 8.37 min, m/z 837.3872 $[M+H]^+$

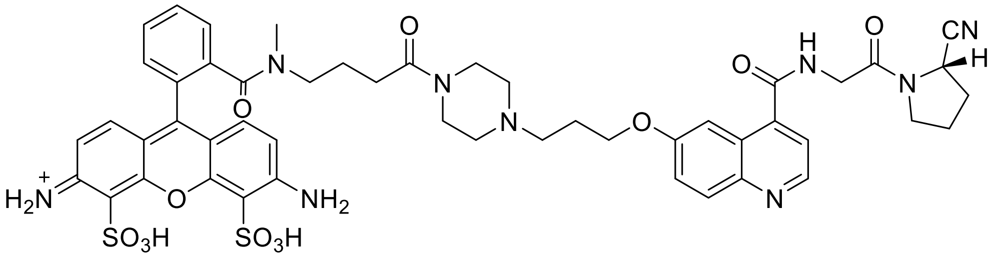

Atto488-FAPI-02 (**14**)

0.66 mg (1.20 µmol) of **11** were treated with 1.33 mg (6.96 µmol) 4-methylbenzenesulfonic acid monohydrate in 250 µL acetonitrile at 45 °C for 4 hours. After removal of the solvent the residue was dissolved in 95 µL dimethylformamide and 5 µL (3.65 mg; 36.1 µmol) triethylamine. 0.54 mg (0.55 µmol) Atto 488 NHS-ester in 25 µL DMSO were added. After 60 minutes 0.49 mg (0.43 µmol; 78%) of the title compound were isolated by HPLC and freeze drying.

LC-MS R_t 10.19 min, m/z 1022.2706 $[M]^+$

Compound Analysis

Reverse-phase high-performance liquid chromatography (RP-HPLC) was conducted using linear gradients of acetonitrile in water (0-100% acetonitrile in 5 min; 0.1% TFA; flowrate 2 mL/min) on a Chromolith Performance RP-18e column (100 × 3 mm; Merck KGaA Darmstadt, Germany). UV-absorbance was detected at 214 nm. An additional γ-detector was used for the HPLC-analysis of radioactive compounds. HPLC-MS characterization was performed on an ESI mass spectrometer (Exactive, Thermo Fisher Scientific, Waltham, MA, USA) connected to an Agilent 1200 HPLC system with a Hypersil Gold C18 1.9 µm column (200 × 2.1 mm; 0-100% acetonitrile in 20 min; flowrate 200 µL/min). Analytical Radio-HPLC was performed using a Chromolith Performance RP-18e column (100×3mm; Merck; 0-30% acetonitrile in 10 min; flowrate 2 mL/min). HPLC-purifications were performed on a LaPrep P110-System (Knauer, Berlin, Germany) and a Reprosil Pur 120 column (C18-aq 5 µm 250 × 25mm; Dr. Maisch, Ammerbuch-Entringen, Germany). The water/acetonitrile-gradient (15 or 25 min; 0.1% TFA; flowrate 20 mL/min) was modified for the individual products.

Radiochemistry

Radioiodine (I-125) was purchased from Hartmann Analytik (Göttingen, Germany); radioactive lutetium (Lu-177) was obtained from ITG (München, Germany); radioactive gallium (Ga-68) was eluted from a Ge-68/Ga-68 generator purchased from Themba Labs (Somerset West, South Africa). F-18-FDG was provided by the ZAG Zyklotron AG (Eggenstein, Germany).

For iodinations 10 µL of the organotin precursor of FAPI-01 (1 µmol/mL in ethanol) were diluted with 10 µL of 1 M HCl and 10 µL water before 1-20 MBq iodine-125 in 0.05 M NaOH were added. The reaction was started by addition of 5 µL of a fresh 1.9% solution of peracetic acid in glacial acetic acid. After 60 s 15 µL of 1 M NaOH were added and the reaction was quenched by addition of 5 µL of 5% ascorbic acid in water before HPLC purification. The obtained solution was directly used for *in vitro* experiments or evaporated to dryness under reduced pressure and taken up in 0.9% NaCl (Braun, Melsungen, Germany) in case of animal studies.

Lu-177 labeling of DOTA-compounds was performed by addition of 5 MBq lutetium chloride to 100 µL of a 10 µM solution of the individual precursor in 0.1 M NaOAc (pH 5) and incubation at 95 °C for 10 min. The solution was directly used for *in vitro* experiments or diluted with 0.9% NaCl (Braun, Melsungen, Germany) in case of animal studies.

Labeling with Ga-68 for animal studies was performed by incubating 255 µL generator eluate (0.6 M HCl; approx. 230 MBq) with a mixture of 1 nmol DOTA-precursor, 1 µL of 20% ascorbic acid in water and 72 µL NaOAc (2.5 M) at 95 °C for 10 min. Remaining free radioactivity was removed by dilution with 2 mL water, solid phase extraction (sep-pak light C18, Waters), washing with 2 mL water and elution of the product with 1 mL water/ethanol 1:1. The obtained solution was evaporated to dryness under reduced pressure and the residue taken up in 0.9% NaCl (Braun).

For determination of the stability in human serum the radiolabeled compounds (approx. 2.5 MBq for I-125 or 15 MBq for Lu-177) were purified (HPLC or solid phase extraction) and freed from solvent. The residues were taken up in 250 µL human serum (Sigma-Aldrich) and incubated at 37 °C. Samples were precipitated with 30 µL acetonitrile and analyzed by HPLC (0-30% acetonitrile in 10 min).

Cell Lines

In vitro binding studies were performed using the human tumor cell lines BxPC3, Capan-2 (both pancreatics adenocarcinoma), MCF-7 (breast cancer), purchased from Sigma Aldrich Chemie GmbH

and SK-LMS-1 (vulva leiomyosarcoma, purchased from ATCC) as well as stably transfected FAP-cells. The human fibrosarcoma cell line HT-1080 has been transfected with the human FAP gene (HT-1080-FAP), whereas HEK 293 cells were transfected with the murine FAP (HEK muFAP) and the CD26 gene (HEK CD26), respectively (transfected cell lines were obtained from Stefan Bauer, NCT Heidelberg (*3*)). All cells were cultivated in Dulbecco's modified Eagle's medium (DMEM) containing 10% fetal calf serum at 37°C/5% carbon dioxide.

Cell Staining and Microscopy

For internalization experiments HT-1080-FAP and HEK muFAP cells were seeded on uncoated coverslips in a 24-well plate and cultivated in culture medium containing 10% fetal calf serum to a final confluence of approx. 80-90%. The medium was removed and cells were washed with 0.5 mL PBS pH 7.4 for 2 times. FAPI-02-Atto488 (20 µM in DMEM) was added to the cells and incubated for 2 hrs at 37°C. Cells were washed with 0.5 mL PBS pH 7.4 for 3 times and fixed with paraformaldehyde (2% in PBS) for 15 min. The overgrown coverslips were placed on microscope slides using mounting medium containing DAPI for cell nucleus staining (Fluoroshield, Sigma-Aldrich). Images were acquired on a laser scanning confocal microscope (Zeiss LSM 700; Zeiss, Oberkochen, Germany) using the Zeiss Plan-Apochromat 63x/1.4 Oil DIC III immersion objective at xy pixel settings of 0.099 x 0.099 µm and 1 Airy unit pinhole size for each fluorophore used (488 nm for FAPI-02-Atto488, 405 nm for DAPI). The pictures were processed consistently using the ZEN 2008 software and ImageJ.

Radioligand Binding Studies

For radioligand binding studies, cells were seeded in 6-well plates and cultivated for 48 h to a final confluence of approx. 80-90% (1.2 - 2 mio cells/well). The medium was replaced by 1 mL fresh medium without fetal calf serum. The radiolabeled compound was added to the cell culture and incubated for different time intervals ranging from 10 min to 24 h. Competition experiments were performed by simultaneous exposure to unlabeled (10^{-5} M to 10^{-9} M) and radiolabeled compound for 60 min. For efflux experiments, radioactive medium was removed after incubation for 60 min and replaced by non-radioactive medium for time intervals ranging from 1 to 24 h. In all experiments, the cells were washed with 1 mL phosphate-buffered saline pH 7.4 for 2 times and subsequently lysed with 1.4 ml lysis buffer (0.3 M NaOH, 0.2% SDS). Radioactivity was determined in a γ-counter (Cobra II, Packard), normalized to 1 mio cells and calculated as percentage of the applied dose (%ID). Each experiment was performed 3 times, and 3 repetitions per independent experiment were acquired.

For internalization experiments the cells were incubated with the radiolabeled compound for 60 min at 37 °C and 4 °C. Cellular uptake was terminated by removing medium from the cells and washing 2 times with 1 mL PBS. Subsequently, cells were incubated with 1 mL of glycine-HCl (1 M in PBS, pH 2.2) for 10 min at room temperature to remove the surface bound activity. The cells were washed with 2 mL of ice-cold PBS and lysed with 1.4 mL of lysis buffer to determine the internalized fraction. For the cells incubated at 4 °C, all washing and elution steps were carried out using ice-cold buffers. The radioactivity was measured using a γ-counter, normalized to 1 mio cells and calculated as percentage of applied dose (%ID).

PET Imaging and Biodistribution Analysis in Mice

All experiments were performed in accordance with the German animal protection laws and complied with European Commission regulations for the care and use of laboratory animals. The mice were anaesthetized using isoflurane inhalation.

For *in vivo* experiments, 8 week old BALB/c *nu/nu* mice (Charles River) were subcutaneously inoculated into the right trunk with 5 × 10^6 HT-1080-FAP or Capan-2 cells, respectively. When the size of the tumor reached approximately 1 cm^3, the radiolabeled compound was injected via the tail vein (∼10 MBq for small-animal PET imaging; ∼1 MBq for organ distribution). For organ distribution of ^{131}I-FAPI-01 and ^{177}Lu-FAPI-02, the animals (n = 3 for each time point) were sacrificed after indicated time points (from 30 min to 24 h). The distributed radioactivity was measured in all dissected organs and in blood using a γ-counter (Cobra Autogamma, Packard). The values are expressed as percentage of injected dose per gram of tissue (%ID/g). PET imaging was performed using the small-animal PET scanner Inveon PET (Siemens). After a 15 min transmission scan the anaesthetized mice were injected with approximately 1 nmol ^{68}Ga-FAPI-02 (∼10 MBq). Within the first 60 min a dynamic scan was performed in list mode, followed by a static scan from 120 to 140 min after injection. Images were reconstructed iteratively using the 3D-OSEM+MAP method (Siemens) and were converted to standardized uptake value (SUV) images. Quantification was done using a ROI technique and expressed as SUV mean.

For pharmakokinetic modeling the transport constant K1 and the rate constants k2–k4 were calculated using a two-tissue compartment model implemented in the PMOD software (*4*), taking into account the vascular fraction (vB), which is associated with the volume of blood exchanging with tissue in a VOI. The rate constants that describe the compartmental fluxes include k1 (binding to the receptor), k2 (detachment) as well as k3 (internalization) and k4 (efflux) in the tumor tissue. In this model the fractional volume of distribution (DV=K1/k2) is the proportion of the region of interest in which ^{15}O-labelled water is distributed.

Data Analysis

Data were analysed using GraphPad Prism 4 (GraphPad Software, San Diego, USA). Data from radioligand binding assays and animal studies are reported as means ± SD unless otherwise indicated.

RESULTS

Serum Stability

Processed and solvent free radioactive compounds (^{125}I-FAPI-01 and ^{177}Lu-FAPI-02) were incubated in human sera at 37 °C. After the respective incubation time samples were taken, freed from proteins by precipitation with acetonitrile, centrifuged and the supernatant analyzed via radio-HPLC. Suppl. Figure 1 shows that even at 24 h only the initial (radioactive) peaks are detected and neither radioactive degradation products nor free radioactivity are observed. These findings indorse, that both substances are unhampered by enzymatic components of human sera.

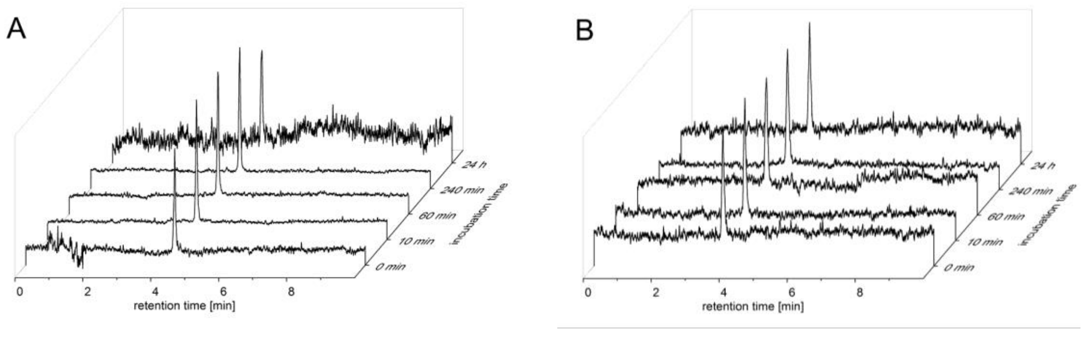

SUPPLEMENTAL FIGURE 4. Stability in human serum of A) FAPI-01 and B) FAPI-02.

Enzymatic Deiodination of FAPI-01

To evaluate time-dependent enzymatic deiodination of ^{125}I-FAPI-01, the cell-based internalization assay was performed at 4°C and 37°C, respectively. As shown in Suppl. Figure 2, FAP-α specific cell binding and internalization of the ligand at 4°C is increasing steadily up to 4 h of incubation whereas incubation at 37°C results in decreasing activities. There results demonstrate a robust deiodination of FAPI-01 due to enzymatic activity, which is inhibited by temperature decrease.

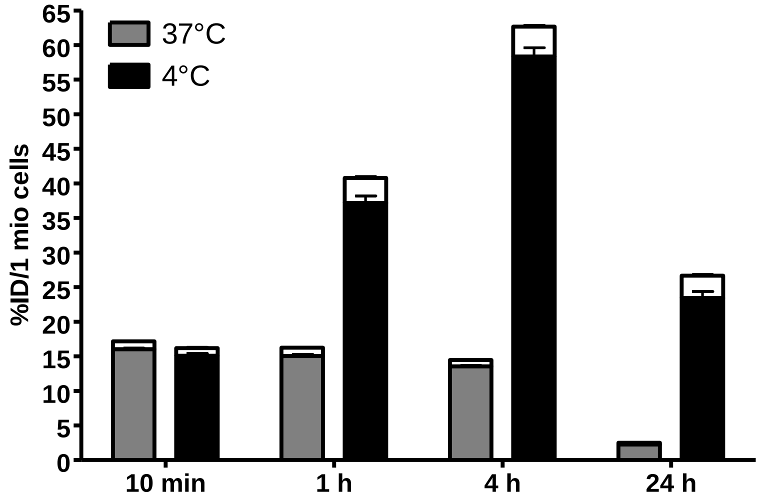

SUPPLEMENTAL FIGURE 5. Internalization of ^{125}I-FAPI-01 into HT-1080 FAP cells at 37 and 4°C.

Pharmacokinetic Analysis of FAPI-02

	Unit	Capan-2 - comp.	HT-1080-FAP - comp.	HT-1080-FAP + comp.
**vB**	l/l	0.08	0.04	0.04
**k1**	ml/ccm/min	0.08	0.07	0.10
**k2**	l/min	0.16	0.13	0.32
**k3**	l/min	0.08	0.10	0.04
**k4**	l/min	0.05	0.02	0.07
**Vs**	ml/ccm	0.93	2.31	0.18
**Vt**	ml/ccm	1.44	0.87	0.48
**Flux**	ml/ccm/min	0.03	0.03	0.01
**Chi²**	----	0.10	0.11	0.26

SUPPLEMENTAL TABLE 1. Pharmakokinetic characteristics of ^{68}Ga-FAPI-02, calculated from dynamic PET data using a two-tissue compartment model according to (*4*). vB: vascular fraction, associated with the volume of blood exchanging with tissue in a VOI (volume of interest); k1-k4: calculated rate constants; Vs: ratio of specific binding concentration to total parent at equilibrium; Vt: total distribution volume.

	Capan-2	HT-1080-FAP
**Blood**	0.83 ± 0.127	1.20 ± 0.178
**Brain**	0.05 ± 0.010	0.06 ± 0.006
**Heart**	0.37 ± 0.031	0.56 ± 0.085
**Intestines**	0.30 ± 0.064	0.37 ± 0.046
**Kidneys**	1.45 ± 0.106	1.60 ± 0.075
**Liver**	0.36 ± 0.015	0.45 ± 0.074
**Lungs**	0.72 ± 0.021	1.02 ± 0.152
**Muscle**	0.94 ± 0.168	1.17 ± 0.332
**Spleen**	0.25 ± 0.015	0.38 ± 0.051
**Tumor**	3.82 ± 0.390	4.51 ± 0.816

SUPPLEMENTAL TABLE 2. Quantification of biodistribution data 1 h after intravenous administration of Lu-177 labeled FAPI-02 to tumor bearing Balb/c nude mice; n=3.

Tumor-to-tissue Ratios of FAPI-02 after Intravenous Administration of Radiolabeled Compound to Tumor Bearing Nude Mice

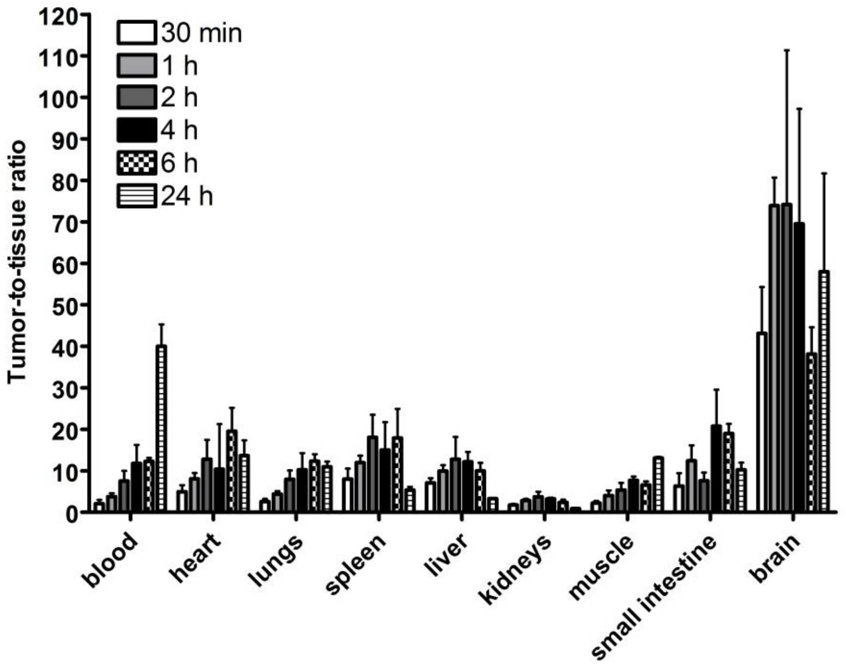

SUPPLEMENTAL FIGURE 6. Tumor-to-normal tissue ratios 30 min to 24 h after intravenous administration of Lu-177 labeled FAPI-02 to HT-1080-FAP tumor bearing Balb/c nude mice; n=3.

Standardized Uptake Values of FAPI-02 after Intravenous Administration to Cancer Patients

SUV max (1h)	Patient 1<br>Pancreatic carcinoma	Patient 2<br>Mammary carcinoma
**Aorta**	2.7	3.6
**Brain**	0.1	0.3
**Ileum**	1.0	1.9
**Liver**	3.0	2.6
**Lungs**	0.9	0.9
**Muscle**	1.0	2.1
**Pancreas**	---	3.4
**Renal parenchyma**	3.9	6.1
**Spleen**	1.9	2.6
**Primary tumor**	10.0	---
**Metastases (average)**	7.6	13.3

SUPPLEMENTAL TABLE 3. Maximum tissue uptake (SUV max) 1 h after intravenous administration of ^{68}Ga-FAPI-02 to patients with metastasized pancreatic and mammary carcinoma.

REFERENCES

1. Mier W, Hoffend J, Kramer S, et al. Conjugation of DOTA using isolated phenolic active esters: the labeling and biodistribution of albumin as blood pool marker. *Bioconjug Chem.* 2005;16:237-240.

2. Jansen K, Heirbaut L, Cheng JD, et al. Selective Inhibitors of Fibroblast Activation Protein (FAP) with a (4-Quinolinoyl)-glycyl-2-cyanopyrrolidine Scaffold. *ACS Med Chem Lett.* 2013;4:491-496.

3. Fischer E, Chaitanya K, Wuest T, et al. Radioimmunotherapy of fibroblast activation protein positive tumors by rapidly internalizing antibodies. *Clin Cancer Res.* 2012;18:6208-6218.

4. Burger C, Buck A. Requirements and implementation of a flexible kinetic modeling tool. *J Nucl Med.* 1997;38:1818-1823.

Author contributions: The project was initiated and supervised by UH. All experiments were designed and conceived by UH, AL, TL and AA. Chemical synthesis and purification of the compounds was performed by TL, radiolabeling of the compounds was done by TL, AL and WM. Radioligand

binding studies, fluorescence staining and animal experiments were performed by AL. NMR analysis of FAPI-02 was provided by PB and CR. Statistical evaluation of the experiments was performed by UH and AL. Clinical examinations were initiated and supervised by UH, CK, FG, JD and DJ. The manuscript was designed by UH, AL and TL. It was written by AL, UH and TL with support from AA and JD.
